# Mimicry and expressiveness of an ECA in human-agent interaction: familiarity breeds content!

**DOI:** 10.1186/s40469-016-0008-2

**Published:** 2016-06-10

**Authors:** Catherine J. Stevens, Bronwyn Pinchbeck, Trent Lewis, Martin Luerssen, Darius Pfitzner, David M. W. Powers, Arman Abrahamyan, Yvonne Leung, Guillaume Gibert

**Affiliations:** 1 MARCS Institute for Brain, Behaviour & Development, Western Sydney University, Locked Bag 1797, Penrith, NSW 2751 Australia; 2 School of Social Sciences & Psychology, Western Sydney University, Penrith, Australia; 3 Informatics and Engineering, Flinders University, Adelaide, Australia; 4 School of Business, Charles Darwin University, Darwin, Australia; 5 Psychology Department, Neurosciences Institute, Stanford University, Stanford, USA; 6 INSERM, U846, 18 avenue Doyen Lépine, 69500 Bron, France; 7 Université de Lyon, Université Lyon 1, 69003 Lyon, France

**Keywords:** Avatar, Chameleon effect, Emotion, Evaluation, Expression, Intelligibility, Likeability, Mirroring, Talking head

## Abstract

**Background:**

Two experiments investigated the effect of features of human behaviour on the quality of interaction with an Embodied Conversational Agent (ECA).

**Methods:**

In Experiment 1, visual prominence cues (head nod, eyebrow raise) of the ECA were manipulated to explore the hypothesis that likeability of an ECA increases as a function of interpersonal mimicry. In the context of an error detection task, the ECA either mimicked or did not mimic a head nod or brow raise that humans produced to give emphasis to a word when correcting the ECA’s vocabulary. In Experiment 2, presence versus absence of facial expressions on comprehension accuracy of two computer-driven ECA monologues was investigated.

**Results:**

In Experiment 1, evidence for a positive relationship between ECA mimicry and lifelikeness was obtained. However, a mimicking agent did not elicit more human gestures. In Experiment 2, expressiveness was associated with greater comprehension and higher ratings of humour and engagement.

**Conclusion:**

Influences from mimicry can be explained by visual and motor simulation, and bidirectional links between similarity and liking. Cue redundancy and minimizing cognitive load are potential explanations for expressiveness aiding comprehension.

**Electronic supplementary material:**

The online version of this article (doi:10.1186/s40469-016-0008-2) contains supplementary material, which is available to authorized users.

## Background

Interacting with ECAs provides a highly controllable medium for the investigation of interpersonal and social behaviour. Additionally, the recovery of social psychology effects using an ECA is an indirect method of evaluation. If an ECA or robot is regarded and treated in human-like ways, then it should be possible to observe phenomena such as social inhibition of return, the social Simon effect (Stenzel et al. 2012), chameleon effect (Bailenson and Yee 2005), and effects of non-verbal cues on trust (DeSteno et al. 2012; Lee and Brezeal 2010). Experiment 1 investigates the hypothesis that behavioral mimicry increases affinity and liking. Experiment 2 examines the benefits for comprehension when an ECA uses expressive facial gestures in telling a story.

## Non-conscious mimicry, similarity and liking

Non-conscious mimicry refers to unintentionally copying another individual’s behaviors, such as postures, mannerisms, facial expressions, speech patterns and emotions (Chartrand and Bargh 1999; Lakin et al. 2003). Non-conscious mimicry has been referred to as a kind of social glue that binds us together as it is thought to be both a cause and an effect of liking an individual (Guéguen and Martin 2009). Similarity is thought to be a key factor in non-conscious mimicry. Through the process of non-conscious mimicry, individuals are able to take on the gestures and mannerisms of the other person, allowing them to increase their similarity to another (Castelli et al. 2009). It is well established that when people perceive themselves as similar to another person they will like the other person more (Nass and Moon 2000; Heine et al. 2009). In the context of an avatar, for example, women have been shown to prefer to interact with an avatar of the same gender (Ruttkay et al. 2004). Thus mimicry fosters social contact; increases similarity and adaptation which permit social bonding (Krämer et al. 2013). In short, rapport builds mimicry and mimicry builds rapport or, as Zajonc (1968) quipped, familiarity breeds content!

A relationship between mimicking and liking has been observed in human-robot interaction. For example, a robot that resembled an ape either mimicked or did not mimic the participants, and participants reported liking the mimicking robot more than the non-mimicking robot (Riek and Robinson 2008). Similarly, a virtual agent that mimicked participant’s head movements while providing information about campus security was judged as more likeable than a non-mimicking agent (Bailenson and Yee 2005). In situations where levels of similarity are extremely low, counter mimicry may occur. For example, there are instances of people smiling in response to another’s wincing (Bourgeois and Hess 2008; Yabar et al. 2006).

More recently, the degree to which humans mimic an agent has been investigated as a means to explore the social connection between humans and a virtual embodied agent (Mattheij et al. 2015). Vocal pitch and affective facial expressions of the agent were manipulated and subsequent vocal and facial expressions of users recorded. Analyses suggest vocal and facial mimicry by users with the results interpreted as signs of unconscious affect recognition and social bonding.

Mimicry in the form of repeating words from a previous speaker-turn has been shown to positively impact subjective reports of user engagement (Campano et al. 2015). The ECA’s behavior was also rated as more believable when repeating words or uttering “other-repetitions”. Back-channeling by an ECA, on the other hand, had little effect on self-report ratings of user engagement (Cavedon et al. 2015). Yaghoubzadeh et al. (2015) treat communication and human-agent interaction as forms of social collaboration and cooperation. Their system is sensitive and responsive to state of both the interaction and the user.

Positive effects of mimicry were evident in an experiment that compared an agent that did not smile, showed occasional smiles, or displayed frequent smiles. Manipulation of the frequency of smiling had no impact on evaluation of the agent but did elicit longer smiles from the user when the agent smiled (Krämer et al. 2013). The results of studies so far suggest subtle, possibly non-conscious mimicry and potential disjunction between behavior and self-report ratings. In Experiment 1, we take a direct approach with the agent mimicking specific features of the human participant, assessment of the human mimicking the agent and, in addition to these implicit, behavioural measures, the collection of self-report ratings.

A question that arises when investigating mirroring or mimicking is the veracity between the original behavior/gesture and the mimicked version. Do they need to be identical, for example (Caridakis et al. 2007; Castellano et al. 2012), and do they need to be consistently recognized by the user (Luo et al. 2013)? Castellano et al. examined copying behaviour with an emphasis on the expressive level, i.e., the quality of the motion rather than higher order semantic relations. A perception experiment revealed that participants were able to associate emotional content of gesture with the expression intended by the user. Similarly, Caridakis et al. noted that mimicry needs to be an expressive model not an exact duplicate of the original behaviour. Luo et al. showed that while people have preference for motions similar to their own, their self-awareness did not impact preference.

## Visual prominence cues

Non-verbal cues, such as visual prominence cues, are ideal for studying non-conscious mimicry. People use visual prominence cues to add emphasis in their speech. For example, Cvejic et al. (2010) used an error correction task in which an animated computer head or a human displayed on a screen said a sentence but with one of the words replaced with an incorrect word. Participants used visual prominence cues to emphasize the incorrect words. In an earlier study, Dutch and Italian subjects preferred eyebrow movement to coincide with the most prominent word in a sentence (Krahmer and Swerts 2004). Eyebrow movement can serve as an independent cue to prominence even though some interplay between visual and acoustic cues to prominence have been shown (Granstrom and House 2005). Eyebrow raises and head nods are effective in emphasizing the prominence of a word (Flecha-Garcia 2010; Munhall et al. 2004). Head nods were associated with the attribution of agreement in a study by Bevacqua et al. (2010).

Generic visual prominence cues, head nod and brow raise, will be used in Experiment 1 as approximations to gestural emphases that users make when repeating a spoken phrase to the agent in a language-learning, specifically error detection, scenario. Experiment 1 will investigate the broad hypothesis that mimicry is associated with judgments of liking and lifelikeness. Is there an increase or decrease, for example, in overall satisfaction with the ECA interaction with greater use of gestural cues? Rating scales will be used to differentiate participants according to how much they like aspects of the ECA and to examine whether there is any relationship between the number of prominence cues users produce, their ratings of the ECA, and the tendency *for participants* in the final phase of the experiment *to mimic the ECA*.

## Aim, design and hypotheses

The aim of Experiment 1 was to investigate the effects of mimicry on liking in human-ECA interaction. The one-way experimental design (repeated measures) had all participants experiencing both the mimic and no-mimic conditions blocked and counterbalanced across the sample. The first hypothesis tests whether those whose visual prominence cues are mimicked display more or less prominence cues than those in the non-mimicking condition. Second, we hypothesized that there is a relationship between the number of prominence cues that users produce and their ratings of an ECA. Finally, we ask, is there an association between the number of prominence cues produced in the mimic condition and the tendency for users to mimic the ECA in the final phase of interaction?

## Method

### Participants

The sample consisted of 40 female participants ranging in age from 18 to 35 years (*M* = 23.33 years, *SD* = 7.05) with normal hearing, normal or corrected to normal vision, and fluent in English. The sample was restricted to females as it is known that sex of user and agent have the potential to interact (e.g., Payne et al. 2013; Ruttkay et al. 2004). Rather than reducing statistical power, sex was controlled. Moreover, in a pilot study, female participants also gave reliable and unambiguous visual prominence cues during an error detection task. The sample was recruited from 1^st^ year psychology students at Western Sydney University. None had experience with programming avatars or knowledge of Wizard of Oz designs.

### Stimuli

The task required participants to say sentences to the ECA, and have these sentences repeated back to them. The sentences were drawn from the IEEE Subcommittee on Subjective Measurements (1969), which comprised lists of phonetically balanced sentences. The three sentence lists used in the current study were chosen for their ease of pronunciation and the relatively small number of syllables which helped participants better recall them when a sentence needed to be re-stated to the ECA (Appendix 1). There were 3 sentence lists, each consisting of 10 sentences. The order of the 3 sentence lists was fully counterbalanced. In addition, the presentation of the sentences (either backwards or forwards) was partially counterbalanced.

Participants read these sentences aloud and one at a time to the ECA; a talking, moving representation of a human head displayed on a computer screen. The ECA was programmed to be able to make a number of different gestures in response to the participants’ speech. The ECA repeated the sentences to the participant, and the ECA then either mimicked the participant’s eyebrow movement and head nod or not, depending on the experimental condition. As a way to subtly encourage a gesture (visual prominence cue) from the participant, on half of the trials when the ECA repeated the sentence uttered by the participant, one word in the sentence was substituted with a similar but incorrect word. The experimenter was able to choose the position of the head nod or eyebrow raise that the ECA exhibited (towards the beginning, middle or end of the ECA-repeated sentence), depending on where the participant’s gesture had occurred.

Rating scale items included likeability, whether the participant feels positive about the ECA’s behaviors and traits; engagement, or the level of motivation for the participant to spend time with the ECA; naturalness/believability, or whether the ECA’s behaviors match up to those expected by a human conversational partner; and entertainment, the amusement that the ECA provides that is not accounted for by the task employed (Table 1). Items were scored on a five point Likert scale, with 1 being ‘totally disagree’, 2 ‘disagree’, 3 ‘neither agree nor disagree’, 4 ‘agree’ and 5 being ‘totally agree’. The rating scale items were presented after both the mimic and non-mimic conditions.

**Table 1:** Rating scale items

1.	I find the ECA likeable.
2.	I find the ECA engaging.
3.	I find the ECA easy to understand.
4.	I find the ECA life-like.
5.	I find the ECA humorous.
6.	The ECA kept my attention.
7.	I would like to interact with the ECA again.
8.	I enjoyed interacting with the ECA.
9.	I felt as if the ECA was speaking just to me.

### Equipment

**Fig. 1:**
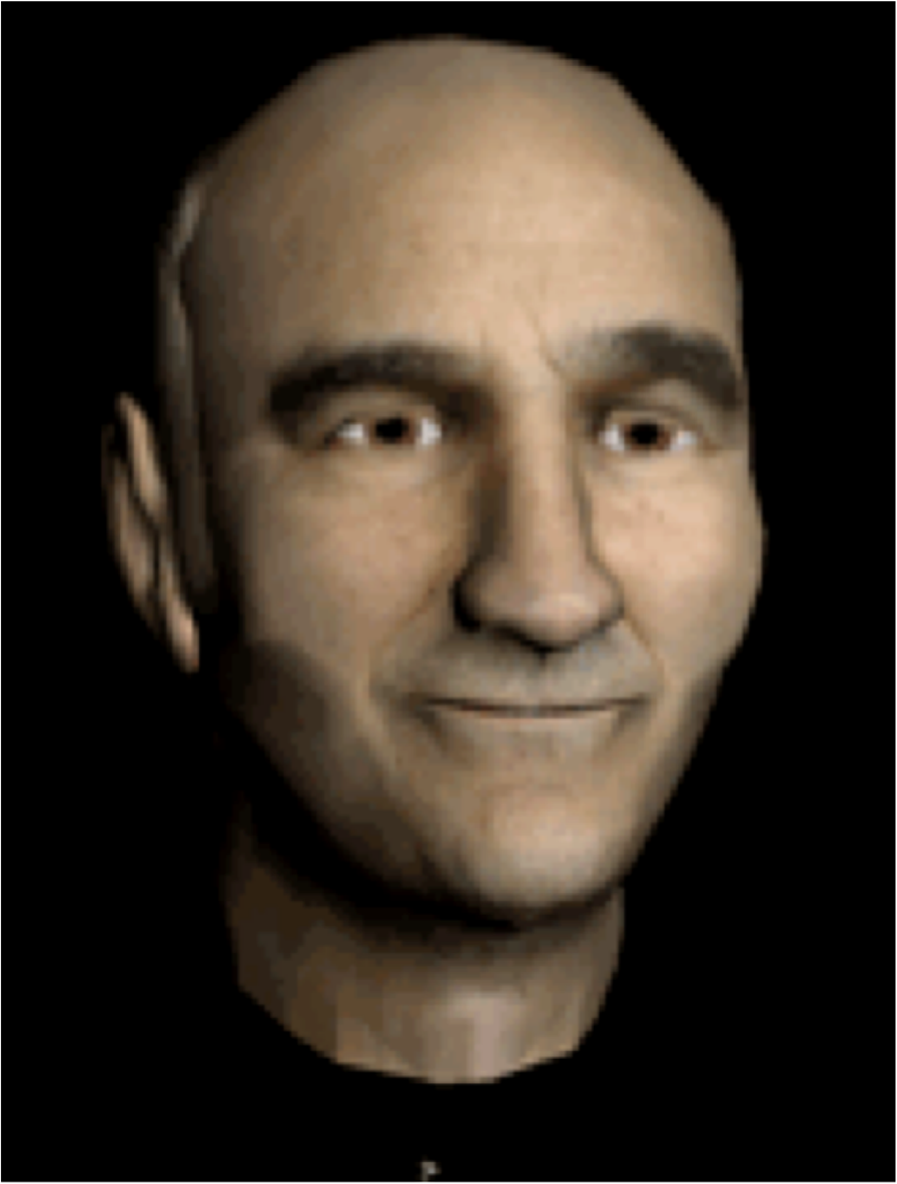
The embodied conversational agent (ECA) used in Experiment 1

The ECA was based on the prosthetic head created in the likeness of performance artist Stelarc (http://stelarc.org). The agent, shown in Fig. 1, was an animated head displayed on an LCD screen which subtended 24.35° visual angle. The visual front-end was a three-dimensional computer-graphic representation of a male face capable of visual speech movements and of displaying basic emotional expressions. The animation component worked as a text-to-AV synthesis system: it received text data intended as speech for the animated face, and generated the speech and corresponding face motion as output. The facial animation was performed by interpolation between a set of 16 visemes; no prephonatory gestures were implemented in this animation model. The system consised of a text-to-speech (TTS) module; a phoneme-to-face motion database; a phoneme-to-face animation generator; and a face animation module (Burnham et al. 2008). The voice of the agent was IBM Viavoice text to speech (TTS) synthesis.

The ECA was displayed on a Cueword Teleprompter (Xpose VGA input monitor) with a colour CCTV video camera (Panasonic WVCL934) installed at the back and a shotgun microphone (Beyer Dynamics MCE86 II) at the side for videorecording. Two laptops (Lenovo T500, Microsoft Window XP Professional v.2002) were connected with a network switch (D-Link 10/100 Fast Ethernet switch) for sending commands from the Event Manager program on one laptop to another which displayed the talking head and sent the image to the teleprompter. The audio sound of the ECA was transferred from the laptop to the USB Audio Capture (EDIROL by Roland UA-25EX) and then sent to the headphones (Sennheiser HD650) and a Ultra Low-noise design 8-input 2-Bus Mixer (Eurorack UB802). The mixer also received audio input from the participants during the recording. It then sent the voice of both ECA (IBM Viavoice text to speech (TTS) synthesis) and the participants to a DV capture device (Canopus ADVC-55) which transferred all the audio input to the recording program (Adobe Premiere Pro 2.0) in a computer. The video camera also sent the recorded images directly to the program.

### Procedure

Participants were recruited for an error detection task, where they were told they would have to detect errors in speech and correct them. They first read an information sheet and provided written consent, including consent to publish, in line with the approval obtained from the Human Research Ethics Committee at Western Sydney University (H7776). On the desk, a laptop displayed Powerpoint slides that contained the selected sentences, shown one at a time. On a teleprompter behind that, participants saw their conversational partner displayed. In the first pilot, participants interacted in real time with the experimenter who was in an adjoining room. In the second pilot and the actual experiment, participants interacted with the ECA in a Wizard of Oz set-up.

Two pilot studies were conducted to determine whether the error correction task could elicit a sufficient amount of eyebrow raises in human participants to be mimicked by the ECA in the experiment. It was also exploratory in that it aimed to discover what non-verbal cues participants exhibited that could be used as a cue to be mimicked. In the first pilot (*N* = 7), participants interacted with the experimenter whose face was projected onto the screen in real time. The task required participants to read one of the prescribed sentences aloud while looking at the screen. The experimenter would then repeat the sentence to the participant. The experimenter’s face remained non-emotive during the reading in order to control for any facial expression effect across participants and eliminate any non-conscious mimicry. The video of the experimenter’s face was checked afterwards. There were ten sentences in total, and five of them contained errors with one of the words replaced. For example, “The pipe ran almost the width of the ditch” instead of “The pipe ran almost the length of the ditch” and “Next Monday is the 12 of the month instead of “Next Sunday is the 12 of the month”. The participant had to recognize the error and repeat the entire sentence correctly. The use of visual prominence cues in uttering the corrected sentence was observed.

For the second pilot (*N* = 5), the design of the experiment was a Wizard of Oz format. In Wizard of Oz studies, the user is led to believe that they are communicating with a fully automated ECA that is capable of recognizing and responding to their speech, emotions and facial expressions. In reality, there is an experimenter, or ‘wizard’ controlling the movements of the ECA (Höök 2004), by pressing the correct button on a computer screen that has a series of pre-programmed speech and gesture cues. The method was the same as in the first pilot. Participants were observed through a live video feed and the ECA was made to repeat the sentences after them. The ECA made some errors that the participants had to correct. As with the first pilot, the participants were exposed to no facial expressions or gestures. The results of the two pilot studies suggested that both head nods and eyebrow raises are used by humans to give prominence to words in correcting a word in a sentence uttered by a human (Pilot 1) or an ECA (Pilot 2).

**Fig. 2:**
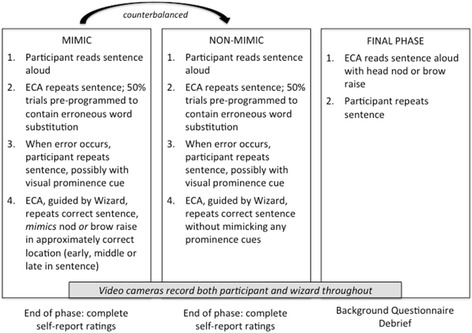
The three phases of Experiment 1; the order of the Mimicking and Non-mimicking ECA was counterbalanced across participants

The main experiment followed a similar format (Fig. 2). Three sets of new, shorter sentences were chosen to replace the longer sentences to help participants remember them so that they could glance at the sentence and then look at the ECA when saying it aloud. (see Appendix 1 for sentence lists). The participants read aloud a sentence and the ECA repeated it. On half of the sentences the ECA was programmed to substitute one of the words with a similar but incorrect word. When an error in vocabulary occurred, the participant repeated the corrected version of the sentence and generally emphasised the correct word with a visual prominence cue. Live video feed of the participant’s facial expressions to the experimenter enabled the experimenter to quickly select a response from a predetermined set of responses, Wizard of Oz style. When participants were in the mimic condition, the experimenter was able to make the ECA mimic the participant’s eyebrow raises *or* head nods at the appropriate place in the sentence (i.e., where the error had occurred) when the ECA repeated the sentence. There was a maximum of one gesture per sentence and 5 in each 10-sentence list had a word substitution error uttered by the ECA. Participants in the non-mimic condition heard the sentences repeated, but were not mimicked. As in Flecha-Garcia (2010), eyebrow movements were defined as any upward movement of the eyebrow.

In the third and final phase of the main experiment, all participants followed the same procedure. However, this time, the ECA (rather than the participant) said the sentence first, and the participant repeated it. The ECA’s sentences were marked up with eyebrow raises and head nods. Participants repeated the sentences after the ECA, and the video of the human was later examined to tally instances of where, in this final phase, the *human* mimicked the ECA.

## Results

Video recordings of participants were coded by two raters and the frequency of visual prominence cues calculated and cross-checked for consistency.

The error correction task elicited visual prominence cues from human users. Comparing sentences that contained errors with those that did not revealed a greater proportion of visual prominence cues produced in sentences with errors spoken by the ECA (*M* = 56 %) than sentences without errors spoken by the ECA (*M* = 35 %).

It was hypothesized that in the first phase of the experiment mimicry by the ECA of the prominence cues made by participants when re-stating a sentence for error correction would increase the number of gestures made by participants. In a one-way repeated measures analysis of variance (ANOVA), there was a main effect of mimicry, *F*(1,38) = 14.30, *p* = .001. However, opposing the hypothesis, the mean number of prominence cues was significantly greater in the ECA non-mimic (*M* = 5.85, *SD* = 2.90) than the mimic condition (*M* = 4.35, *SD* = 2.95).

Correlations were examined to identify the relationship between the number of prominence cues produced and ratings of the ECA. The more prominence cues made, the higher the ratings of ECA lifelikeness in the ECA mimic condition, *r* = 0.26, *p* = .05. A significant negative correlation was evident between the number of prominence cues produced in the ECA non-mimic condition and ratings on humour, *r* = −.305, *p* < .05. This association suggests that the more prominence cues the user produced, the less humourous the ECA was judged to be. There were no other significant correlations.

Finally, we investigated whether the number of ECA gestures mimicked by the user in Phase 3 and the number of prominence cues produced by the user in the mimic condition were correlated, revealing a positive correlation of *r* = 0.274. A positive correlation was also evident between the number of ECA gestures mimicked by the user in Phase 3 and the number of prominence cues they generated on the block of trials just prior to Phase 3, *r* = 0.436. Out of 10 possible ECA gestures to be mimicked in Phase 3, the mean number of ECA gestures mimicked by users was 1.33.

## Discussion

The effect of mimicry on liking in interacting with an ECA was investigated in Experiment 1. An error correction task effectively encouraged production of visual prominence cues from participants with a greater proportion of prominence cues produced after the ECA uttered a sentence erroneously rather than speaking it correctly. Contrasting with the first hypothesis, more visual prominence cues were produced in the condition where the ECA did not mimic the user. Some ratings were correlated with the frequency of producing visual prominence cues. More prominence cues, for example, were associated with greater judged lifelikeness of the ECA in the ECA mimic condition. As hypothesized, there was a tendency for greater mimicry of the ECA by the participant in the final phase when those participants had produced more prominence cues in the mimic condition. Experiment 1 has demonstrated mimicry as a way to enhance gestural similarity between ECA and human that has in turn increased ECA lifelikeness. The absence of a significant positive correlation between amount of mimicry and all self-report ratings of ECA liking could be explained by the non-conscious nature of mimicry.

Current theorizing about the coupling of perception and action begins to explain the dynamical system that forges links between gesture, similarity and enjoyment. For example, Kahl and Kopp (2015) discuss mirroring, mentalizing and social connection. The brain is described as a predictive system with feedforward and inverse models wherein perception guides action and action guides perception. Observation of another is mediated by not only visual but also motor simulation. Understanding is achieved through shared experience and motor simulation or motor resonance. Specifically in Experiment 1, when the ECA makes an error in its speech, the human repeats the sentence and adds prominence to their restatement of the sentence by nodding or raising an eyebrow, among other means of emphasis. The ECA then immediately repeats the correct sentence mimicking those visual gestures of emphasis. Although not necessarily conscious, the expressive intent in the ECA’s gesture is familiar, that is, resonates in the human. And so a system of accommodation and behavioural synchronization, possibly unconscious, takes place with lifelikeness increasing through a sense of familiarity and affinity, bolstering production of visual prominence cues and further mimicry.

Notably, in Experiment 1, the mimic condition relative to the non-mimic condition did not elicit a greater number of visual prominence cues. One explanation for this surprising effect is that prominence cues of the human triggered by the ECA may have taken place immediately after the ECA had mimicked their prominence cues but this was not detected as it took place place when the participant read the sentence that began the *subsequent* trial. A system for coding the accumulation of prominence cues across trials may be a more sensitive measure of human-agent synchronisation and mimicry.

Another possibility for future studies of non-conscious mimicry and liking using ECAs would be to explore other ways to display mimicry. Here, visual prominence cues were chosen as head nods and eyebrow raises could be displayed quickly on the corrected word within a trial. However, in sentence correction, prominence can also be marked by acoustically stressing the correct word, for example, saying the word more loudly, more slowly, or with different intonation. It was not possible to make such acoustic changes online and immediately within a trial. Rather we opted for pre-programmed visual prominence cues that the Wizard operator could select quickly and would be displayed promptly when the ECA repeated the sentence. Other issues include that the fidelity of the visual prominence cues may have been less than ideal. Finally, the assumption underlying mimicry influencing liking and liking influencing mimicry is that mimicry increases similarity between people and increased similarity is associated with liking (e.g., Castelli et al. 2009). The sample in the study was female whereas the ECA had a male face and other effects may be obtained when the gender of the ECA and participants match (e.g., Ruttkay et al. 2004).

The cyclical and dynamic nature of mimicry in interpersonal interaction (Guéguen and Martin 2009) has been demonstrated in the context of human-ECA interaction with lifelikeness reported to be greater when the ECA mimicked participant gestures. A more life-like ECA may not only engender naturalness in human-ECA interaction but also minimize cognitive load and aid comprehension of the ECA’s spoken utterances. This assumption is explored in Experiment 2 by comparing the user’s comprehension accuracy in response to an ECA with marked-up facial expressions compared to one without such expressions.

### Experiment 2 – the effect of visual expressive gestures on comprehension of a spoken ECA monologue

Visual expressiveness of ECAs has become increasingly sophisticated over the last decade (e.g., Campano et al. 2015; Ledge et al. 2015; Lisetti and Hudlicka 2014). Kang and Watt (2013), for example, have demonstrated that psychological co-presence of an avatar and interactant satisfaction are increased with greater avatar anthropomorphism. Nunamaker et al. (2011) demonstrated that, among other things, smiling agents were judged as more likeable.

ECA expressiveness can affect not only user engagement and liking but also comprehension accuracy. Doumanis and Smith (2013) similarly showed the benefits of more realistic depictions of a virtual human on retention of information acquired during an experimental session. The benefits for comprehension, according to the authors, are related to gestures and facial expressions affecting redundancy, which results in more learning. Conrad et al. (2015) manipulated an agent’s dialog capability (high, low) and facial animation (high, low) and examined their effect on users’ comprehension and engagement in completing a survey. Answers to the survey were more accurate in response to agents with high capability dialog and these agents were judged as more personal and less distant. Facial animation did not affect response accuracy but elicited greater engagement and judgments of less naturalness. The crossing of the two factors, dialog and facial animation, has effectively revealed differential effects on survey responses. Context, in short, makes a difference. The authors recommend survey-interviewing agents be designed in light of the nature of the survey questions, i.e., whether the questions refer to sensitive topics or not.

### Aim, design & hypotheses

The aim of Experiment 2 was to investigate the effect of the ECA’s facial expressive gestures, such as smiling, winking, rolling eyes, etc., when reciting a story on measures of story comprehension and agent liking. Logically, a visually expressive ECA has the potential to either enhance or impede user learning and comprehension. The monologue story text was a between-subjects factor and expression within-subjects so that participants acted as their own control, interacting with both an expressive and non-expressive agent. The first measure was accuracy on a 6-item comprehension task with the items relating to the 3-min text that the ECA recited. If expression aids communication, intelligibility and/or engagement, then comprehension performance should reflect that enhancement relative to a condition where there is no facial expression. The second measure, more explicit and direct, involved participants rating five qualities of the ECA: i) Likeable; ii) Engaging; iii) Easy to understand; iv) Life-like; and v) Humorous. At the end of a session, participants assigned ratings across the session to the following statements: i) The ECA kept my attention; ii) I would like to interact with the ECA again; iii) I enjoyed interacting with the ECA; iv) I felt as if the ECA was speaking just to me.

It was hypothesized that if facial expressive gestures aid intelligibility and communication, comprehension accuracy would be greater in response to the expressive than the non-expressive ECA. If it is the case that facial expression increases engagement but greater engagement distracts users from the text, then we would expect a negative correlation between comprehension accuracy and ratings of engagement (i.e., a tendency for lower comprehension scores to be associated with higher engagement scores, and vice versa).

## Method

### Participants

Forty adult participants divided into two groups of 20 and recruited from the 1^st^ year Psychology population at Western Sydney University completed Experiment 2 (33 females and 7 males; *M* = 19.20 years, *SD* = 3.08 years, range 17–35 years). All participants were fluent in English, had normal hearing and normal or corrected to normal vision. None had interacted with an avatar before. Participants received course credit for their participation.

### Stimuli and equipment

Each participant was presented the two texts, one on Edwin Hubble and the other on Machu Picchu (Appendix 2), read aloud by the ECA with one text accompanied by facial expressions and the other text with no facial expressions.

Texts read by the ECA were sourced from abcteach Reading Comprehension worksheets for secondary school (http://www.abcteach.com/directory/reading-comprehension-16-2-1). A pilot test (*N* = 5, *M* = 24.4 years, *SD* = 2.30) was undertaken comparing 10 different texts and multiple choice comprehension items to enable the selection of two texts of comparable length and difficulty. Texts that were too easy (comprehension more than 90 %) or too difficult (comprehension less than 30 %) were discarded. In the pilot test, for example, mean comprehension accuracy in response to Hubble was 63 %. Readability statistics for Hubble and Machu Picchu texts are shown in Table 2. The texts when read aloud took approximately 3 min each.

**Table 2:** Readability statistics for Hubble and Machu Picchu texts

	Hubble	Machu Picchu
Number of words	328	250
Average words per sentence	18.2	17.8
Average characters per word	5.0	4.9
Percentage passive sentences	27 %	57 %
Flesh-Kincaid Reading Ease (out of 100, higher score, greater reading ease)	41.0	48.8
Flesch-Kincaid Grade Level (equivalent to US school grade)	12	10.8

In Experiment 2, the expression factor was crossed with text so that, across the sample, participants were presented with expressive and neutral versions of both texts but an individual participant was presented with just two different texts with one being expressive and the other being neutral. Orders of text and expression were counterbalanced to distribute serial order effects. The five rating scale items with five degrees of responding were: The ECA is: i) likeable; ii) engaging; iii) easy to understand; iv) life-like; and v) humorous. The ECA in Experiment 2 was the Head0+ version of the agent described in Experiment 1. Expressive gestures were marked up manually and the ECA programmed to produce the gesture at the end of a word or phrase.

For Experiment 2 random facial expressions were included at specific points in the dialogue. These facial expressions were drawn from a set of 60 pre-programmed facial motions each of which was composed of between one to seven morph targets out of a potential 25 such as smile, left and right eye brow motions, left and right eye lids motions, eye movements (up/down and left/right), and head movements (up/down and left/right). Various emotive facial expression were defined such as: fear, wink, bliss, longkiss, shortkiss, lemon, surprise, frust, sad, wonder, brows, neutral, anger, grin, shock, absent, yeahright, insulted, and happy.

An example of the full markup for the Hubble text is given in Appendix 3 and Additional file 1. An excerpt from this is: “{BREAK 0.2} {EMOTE random} He studied our galaxy, {BREAK 0.2} {EMOTE random} the Milky Way, {BREAK 0.2} {EMOTE neutral 0.5 random 0.5 neutral 0.8 random 0.8} which consists of ten billion stars, space dust, and gas.” The BREAK tag stops the processing for a period specified in seconds and the EMOTE tag instructs the ECA to produce the facial expression for the specified duration, in seconds. Multiple expressions specified in a single EMOTE tag are queued and played sequentially. Therefore, in the excerpt given the final EMOTE tag will play a neutral expression of 0.5 s, followed by a random expression for 0.5 s followed by a neutral expression for 0.8 s, and finally another random expression for 0.8 s. These expressions were morphed onto the ECA whilst speaking the text “which consists of ten billion stars, space dust, and gas”.

### Procedure

**Fig. 3:**
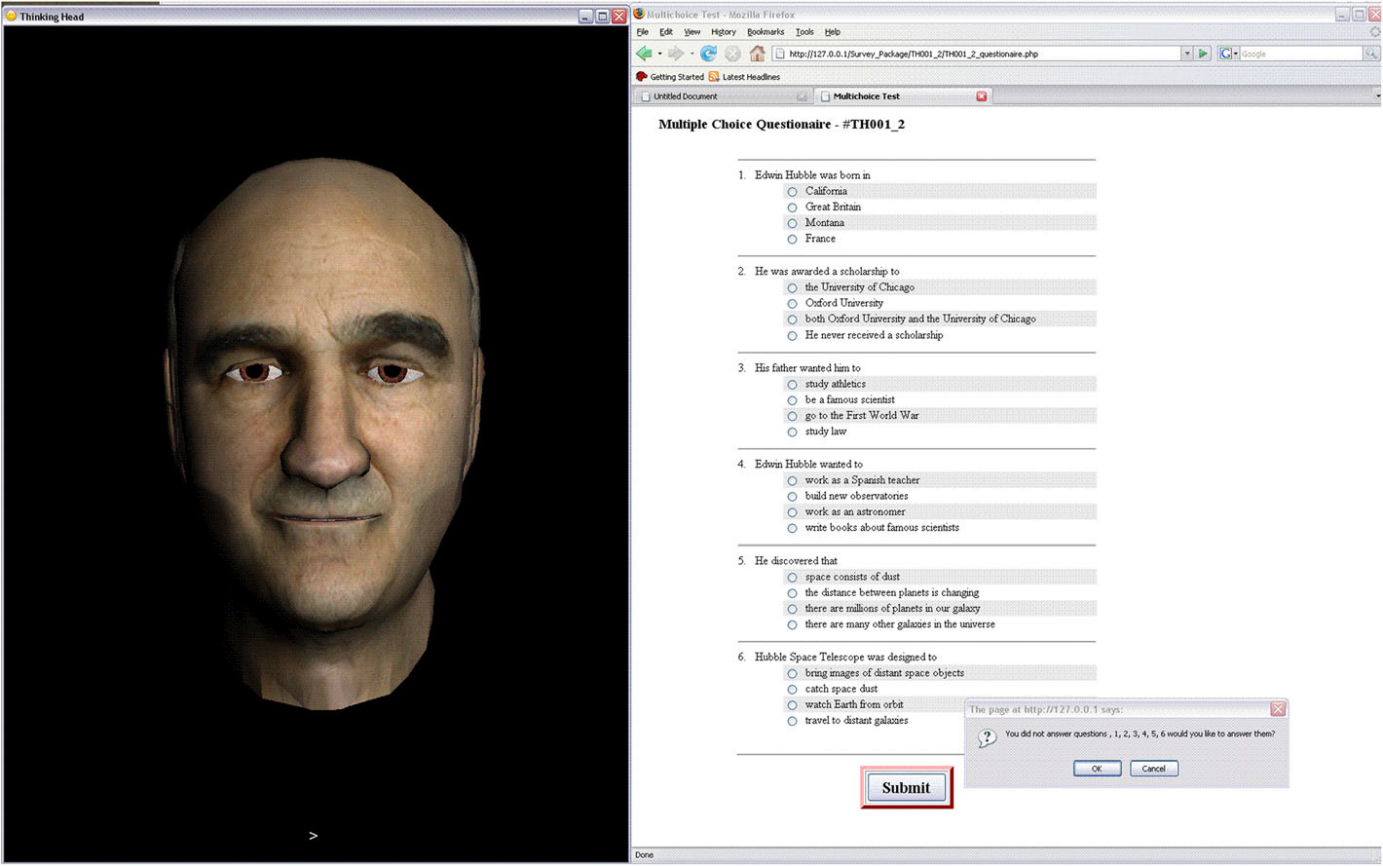
ECA used in Experiment 2. Four-alternative multiple-choice questions for the Hubble story were presented as shown

On arrival, participants read an information sheet and provided written consent, including consent to publish (H7776). Each was presented with the two monologues recited by the ECA with one version of each text accompanied by facial expressions and the other with no facial expression. Experiment sessions were run as groups of three with each participant assigned a PC that was running the ECA software. The experiment stages were as follows. The ECA read one of the texts aloud. Participants then completed the five rating scale items followed by a one-minute break. After the break, participants completed the comprehension task that consisted of six, four-alternative multiple-choice questions about the story, see Fig. 3 for a screenshot. For example, “Edwin Hubble was born in: a) California; b) Great Britain; c) Montana; or d) France”. The rating scale items and the one-minute break provided a short delay between text presentation and retrieval of information from long-term (rather than short-term) memory. Then the second text was presented followed by ratings, a one-minute break, the comprehension task, and then the final set of session ratings.

## Results

Experiment 2 data consisted of the number correct of multiple choice questions for each text, maximum score was 6, reported here as proportions, and response to rating scale items. The results provided no evidence of a negative correlation between comprehension and engagement, suggesting that greater engagement does not necessarily distract the user from the meaning in the text. With that in mind we move to the comprehension scores, the means and standard deviations of which are shown in Table 3.

**Table 3:** Experiment 2: mean comprehension accuracy (as proportions)

	Hubble		Machu	
Condition	Mean	*SD*	Mean	*SD*
Expressive	0.72	0.15	0.52	0.18
Neutral	0.58	0.23	0.54	0.25

There was significantly better comprehension for the Expressive Hubble than the Neutral Hubble text, *t*(19) = 2.22, *p* = 0.04, but not for the Expressive Machu than the Neutral Machu texts, *t*(19) = 0.364, *p* = .72. Thus, there is partial support for the notion that comprehension improves when greater expression is provided. This differential effect is illuminated by the fact that there is better comprehension for the Expressive Hubble than the Expressive Machu text, *t*(19) = 4.17, *p* = .001, but no difference for their neutral counterparts, *t*(19) = 0.55, *p* = 0.59, suggesting that the nature of the mark-up may have affected comprehension.

To investigate text effects more closely, an independent text evaluation task was conducted in which we compared the effect of the two different texts only recited (i.e., no visual information) on comprehension and ratings (*N* = 10, 6 males, 4 females; *M* = 19.60 years, *SD* = 2.22, range 18–24 years). There was no significant difference between the read-aloud Hubble (*M* = 0.75, *SD* = 0.44) and Machu (*M* = 0.70, *SD* = 0.46) texts on comprehension (*p* = .54). The only rating that differed across the texts was the Hubble text was rated as significantly more positive (*M* = 4.00, *SD* = 0.67) than the Machu text (*M* = 3.40, *SD* = 0.52), *t*(9) = 2.71, *p* = .02. As the results from conditions in this audio-only presentation are comparable, it suggests that the nature of the mark-up may have affected comprehension in the auditory-visual version of the experiment.

**Fig. 4:**
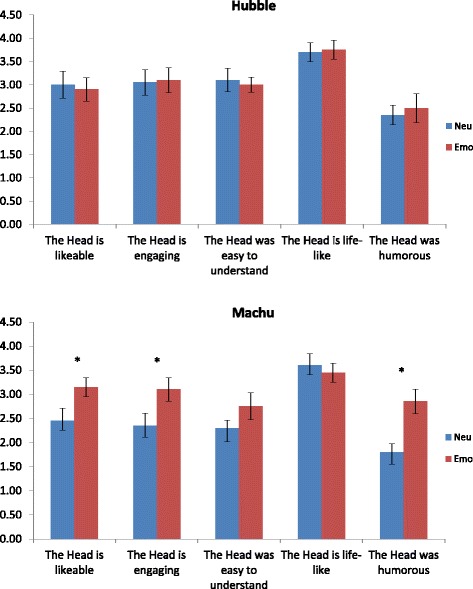
Mean ratings in response to Hubble (*upper*) and Macchu (*lower*) stories as a function of neutral (*blue*) and emotional (*red*) agent expression. Significant effects are marked * (*p* < .05)

Analysis of the participants’ ratings of the ECA showed that the expressive version of the Machu text was perceived to be significantly more humorous than the corresponding Neutral text, *t*(19) = 3.71, *p* = 0.001. There was also a tendency for the Expressive Machu text to result in the ECA being more likeable and engaging than the neutral version of the Machu text (*p* = 0.04). Mean ratings for the Hubble and Macchu stories are shown in Fig. 4. One possibility is that being overtly expressive, and in particular, trying to be funny, or being seen to make fun of the text, detracts from comprehension. To tease these out in future experiments, it is proposed to have separate ratings such as “witty”, “silly” and “serious”.

## Discussion

Experiment 2 examined the effect of visual expressive gestures such as winking, smiling, eye rolling, on story comprehension and subsequent fact recognition. Comprehension was significantly greater for one of the two texts when visual expressive gestures were present. The text difficulty had been matched and an auditory-only version of the experiment revealed a single difference namely that the Hubble text was rated more positively. The results suggest that effectiveness of visual expression on comprehension is influenced by text content. One obvious difference is that the Hubble text was about a person and Machu Picchu about a place.

As ratings of engagement were uncorrelated with comprehension, distraction by a visually more expressive agent does not provide an explanation. Other rating scale items shed some light with ratings of the humour of the Macchu expressive condition being significantly higher than ratings assigned to the neutral Macchu version. The agent conveying the Macchu text with expressive gestures was judged as more likeable and engaging than the neutral version. There is an interaction then between story content, expression and perceived humour.

The results align with recent experiments where greater visual and behavioural realism engenders increased user satisfaction (Kang and Watt 2013) and likeability (Nunamaker et al. 2011). The significant improvement in the Hubble comprehension scores when the agent was expressive could be explained as improved learning when there is greater overlap or redundancy of verbal and visual cues (Nunamaker et al. 2011). In instructional design, for example, the integration of text and graphic can aid understanding, retention and recall of material (e.g., Tindall-Ford et al. 1997). Cue redundancy serves to reduce cognitive load (Stevens et al. 2013). Conrad et al. (2015) reported differential effects of facial animation and dialog capability moderated by context. The present results further corroborate such a relationship and point to the need to design agents and features in tandem with context and content. Other factors at play that have been observed in earlier studies and warranting investigation include the relationship of gender of agent and user (Payne et al. 2013; Ruttkay et al. 2004) and the timing of expressive gesture and spoken content. Simultaneous presentation, for example, may not be the optimal way to convey emotion to the human interlocutor.

### General discussion

In two experiments, features of human to human interaction that are known to influence liking, engagement and learning have been manipulated. Experiment 1 demonstrated the association between agent-user similarity and liking. Non-conscious mimicry of visual prominence cues set up a dynamical, bi-directional exchange between agent and user. Some evidence was obtained suggesting greater lifelikeness of an ECA when mimicry of visual prominence cues has taken place.

Increased social connection may arise from what Kahl and Kopp (2015) term mirroring and mentalizing. Understanding another is achieved not necessarily by only what is said but through shared experience and motor simulation or resonance (Kahl and Kopp 2015). In essence, observation and interaction of a human or agent is mediated by both visual and motor simulation. A visual prominence cue mimicked by an ECA resonates for the user in motor and visual neural networks. Without it necessarily being conscious, such resonance or simulation may drive a feeling of knowing and familiarity. Familiarity can lead to bonding, and bonding to familiarity. Thus a dynamical interplay between agent and user emerges with synchronization and familiarity building social connection and affinity. Remarkably, the mimicked action does not need to be an exact duplicate (Caridakis et al. 2007; Castellano et al. 2012); an expressive model will suffice.

In Experiment 2, the comprehension of a story told by a more visually expressive agent was greater than that of a non-expressive agent. Subtle interactions between facial expression and story content manifested through the use of two stories that were matched in difficulty but differed in content. The more expressive agent tended to be more liked but was also regarded as humorous when the story was less biographical. Expressive facial gestures may aid learning through increased redundancy and increased cognitive load (Stevens et al. 2013; Tindall-Ford et al. 1997). No correlation between comprehension and ratings of engagement rules out distractibility from the more expressive agent as an explanation.

## Conclusion

The two experiments reveal human to human interaction phenomena in human-agent interaction. The results lend some support for the use of visual prominence cues and mimicry as means to build agent preference and trust. Non conscious mimicry of visual (and auditory) cues may further enhance engagement with agents entrusted to collect medical histories, survey people, or provide museum, health, transport or financial information. Both context and content interact. The recovery of human-like interactive behavior legitimizes the use of agents not only in applied settings but as Heyselaar et al. (2015) suggest also as ways to present tightly controlled stimuli and dialogue in pure and applied laboratory experiments examining language, memory, learning, and decision making.

## Appendix 1

### Experiment 1 Sentence lists

Slide the box into that empty space.

The plant grew large and green in the window.

The beam dropped down on the workman’s head.

Pink clouds floated with the breeze.

She danced like a swan, tall and graceful.

The tube was blown and the tire flat and useless.

It is late morning on the old wall clock.

Let’s all join as we sing the last chorus.

The last switch cannot be turned off.

The fight will end in just six minutes.

Open your book to the first page.

Fish evade the net and swim off.

Dip the pail once and let it settle.

Will you please answer that phone.

The big red apple fell to the ground.

The curtain rose and the show was on.

The young prince became heir to the throne.

He sent the boy on a short errand.

Leave now and you will arrive on time.

The corner store was robbed last night.

Nine rows of soldiers stood in line.

The beach is dry and shallow at low tide.

The idea is to sew both edges straight.

The kitten chased the dog down the street.

Pages bound in cloth make a book.

Try to trace the fine lines of the painting.

Women form less than half of the group.

The zones merge in the central part of town.

A gem in the rough needs work to polish.

Code is used when secrets are sent.

## Appendix 2

### Experiment 2 Story Texts

#### Hubble

American astronomer Edwin Hubble was born in November 1889 in Montana. After a few years’ study at the University of Chicago, he was awarded a scholarship to Oxford University in Great Britain for his excellent athletic and academics skills. As he had promised his father, Edwin concentrated on studying law and foreign languages rather than science. When he returned to the United States, he became a teacher of Spanish. However, his love was astronomy. After World War I, he accepted an offer to work in the prestigious Mount Wilson Observatory. Ambitious and energetic, he pursued his career and became the most significant astronomer in the history of cosmology. His research was focused on the universe as a whole. He studied our galaxy, the Milky Way, which consists of ten billion stars, space dust, and gas. In the 1920s, he proved that there are millions of other galaxies, and that the distance between them changes according to certain rules. This break-through, combined with his further discoveries, overthrew the previous theory of a static, or unchanging, universe, and made Edwin Hubble the founder of modern cosmology. His investigations and publications brought him immense recognition, even among ordinary people. Despite this recognition, the Nobel Prize was still only a dream for Edwin Hubble at the time of his death in 1953. Besides our galaxy with its stars and planets, only three other galaxies can be seen without the use of a telescope. More can be seen from observatories, but it is still not enough for many researchers. For this reason, on April 24, 1990, almost a hundred years after Hubble had been born, the National Aeronautics and Space Administration (NASA) launched a huge telescope into space, which they named after him. The Hubble Space Telescope is constructed from large mirrors and lenses that must be perfectly set, a camera, and several satellites transmitting images of distant objects. It helps scientists to study these objects and to understand processes in the universe.

#### Machu Picchu

Machu Pichu (which means "manly peak") is an ancient Incan city located high in the Andes mountains of Peru. It is on top of a ridge between mountains, and is hidden from the Urabamba gorge below. The mighty mountain Huaynac Pichu towers above, and green jungle surrounds the ancient city. It was built between 1460 and 1470 AD by the Incan ruler Pachacuti Inca Yupanqui, and was probably a royal estate and religious retreat. There are about 200 buildings in Machu Pichu. Many of the buildings are homes, but there are also many temples and other buildings, such as storehouses. The buildings are all made of granite blocks that were cut with bronze or stone tools, and then smoothed with sand. Each block fits perfectly against the next block, and no mortar was used! Several hundred years ago, Machu Pichu was abandoned for an unknown reason. With civil war within the Incan Empire, massive deaths because of European diseases, and the Spanish invasion, Machu Pichu was soon forgotten, and even Pizarro, the man who finally conquered the Incas, probably never knew about Machu Pichu. Machu Pichu remained untouched for about four hundred years, before it was rediscovered by Hiram Bingham in 1911. Mr. Bingham was originally looking for Vilcabamba, the last undiscovered stronghold of the Incan Empire. When he found Machu Pichu, he believed it was Vilcabamba. For several years, Machu Pichu was thought to be Vilcabamba, but most people now believe Machu Pichu was an entirely different city.

## Appendix 3

### Emotion Mark-up for the Hubble text

{EMOTE random 0.3 neutral 1 random random neutral 1.8 random 0.25} American astronomer Edwin Hubble was born in November 1889 in Montana. {BREAK} {EMOTE random 0.8 neutral 1.2 random neutral 1.8 random neutral 2 random 2 | neutral 7 random 1} After a few years' study at the University of Chicago, he was awarded a scholarship to Oxford University in Great Britain for his excellent athletic and academics skills. {BREAK}

{EMOTE random neutral 1.5 random 0.4 | neutral 0.5 random} As he had promised his father, Edwin concentrated on studying law and foreign languages rather than science. {BREAK 0.2} {EMOTE neutal 0.3 random 0.5 | neutral 0.8 random 0.2 neutral 0.8 random} When he returned to the United States, he became a teacher of Spanish. {BREAK 0.2} {EMOTE random neutral 0.2 random} However, his love was astronomy. {BREAK 0.2}

{EMOTE random 0.5 | neutral 2 random neutral 1 random} After World War one, he accepted an offer to work in the prestigious Mount Wilson Observatory. {BREAK 0.2} {EMOTE random 0.5 | random 0.3 neutral 1 random 1 neutral 1 random 0.3} Ambitious and energetic, he pursued his career and became the most significant astronomer in the history of cosmology. {BREAK} {EMOTE random} {BREAK}

{EMOTE neutral 3 random} His research was focused on the universe as a whole. {BREAK 0.2} {EMOTE random} He studied our galaxy, {BREAK 0.2} {EMOTE random} the Milky Way, {BREAK 0.2} {EMOTE neutral 0.5 random 0.5 neutral 0.8 random 0.8} which consists of ten billion stars, space dust, and gas. {BREAK 0.2} {EMOTE neutral 4 random 0.1 random 0.1 | neutral 4 random neutral 1 random 1 neutral 1 random 1} In the 1920s, he proved that there are millions of other galaxies, and that the distance between them changes according to certain rules. {BREAK 0.2}

{EMOTE random random} This break-through, combined with his further discoveries, {BREAK 0.1} {EMOTE random | neutral 0.8 random 0.2} overthrew the previous theory of a static, or unchanging, universe, {BREAK 0.2} {EMOTE random 1 | neutral 2.5 random} and made Edwin Hubble the founder of modern cosmology. {BREAK 0.2} {EMOTE neutral 0.5 random 0.7 neutral 0.5 random 1 | random neutral 2.2 random} His investigations and publications brought him immense recognition, even among ordinary people. {BREAK 0.2} {EMOTE random 1 neutral 0.5 random 3 | neutral 1 random 2 neutral 3 random 1}

Despite this recognition, the Nobel Prize was still only a dream for Edwin Hubble at the time of his death in 1953. {BREAK 0.2}{EMOTE neutral 0.3 random 1 | neutral 0.2 random 0.5} Besides our galaxy with its stars and planets, {BREAK 0.2} {EMOTE random neutral 1 random 0.2} only three other galaxies can be seen without the use of a telescope. {BREAK 0.2} {EMOTE random 0.3 | random} More can be seen from observatories, {BREAK 0.2} {EMOTE neutral 0.5 random 1} but it is still not enough for many researchers. {BREAK 0.2} For this reason, {BREAK 0.2} {EMOTE random} on April 24, 1990, {BREAK 0.2} {EMOTE neutral 2 random | neutral 1.5 random 0.2} almost a hundred years after Hubble had been born, {BREAK 0.2} {EMOTE neutral 5.4 random} the National Aeronautics and Space Administration (NASA) launched a huge telescope into space, which they named after him. {BREAK} {EMOTE random} {BREAK} {EMOTE random neutral 1 random 0.3 neutral 4 random 0.2} The Hubble Space Telescope is constructed from large mirrors and lenses that must be perfectly set, {BREAK 0.1} a camera, {BREAK 0.1} {EMOTE neutral 0.5 random | neutral 0.2 random 0.3} and several satellites transmitting images of distant objects. {BREAK 0.2} {EMOTE random neutral 0.5 random} It helps scientists to study these objects and to understand processes in the universe. {BREAK 0.2} {EMOTE random}

## Additional file

Additional file 1: Code and full markup of Hubble text - Expressive condition. (PDF 28 kb)
