# Supplementary material for: Mimicry and expressiveness of an ECA in human-agent interaction: familiarity breeds content!
Source: Comput Cogn Sci. 2016 Jun 10;2(1):1. doi: 10.1186/s40469-016-0008-2 (PMC5125404; doi:10.1186/s40469-016-0008-2)
Supplement: Additional file 1: — Code and full markup of Hubble text - Expressive condition. (PDF 28 kb) [file 40469_2016_8_MOESM1_ESM.pdf]

```
  <!--SURVEY-->

  <category>
    <pattern>STXPNH</pattern>
    <template>
      American astronomer Edwin Hubble was born in November 1889 in
Montana. {BREAK 0.2} After a few years' study at the University of
Chicago, he was awarded a scholarship to Oxford University in Great
Britain for his excellent athletic and academics skills. {BREAK 0.2}

      As he had promised his father, Edwin concentrated on studying
law and foreign languages rather than science. {BREAK 0.2} When he
returned to the United States, he became a teacher of Spanish.
{BREAK 0.2}

      However, his love was astronomy. {BREAK 0.2} After World War
one, he accepted an offer to work in the prestigious Mount Wilson
Observatory. {BREAK 0.2} Ambitious and energetic, he pursued his
career and became the most significant astronomer in the history of
cosmology.  {BREAK 0.2}

      His research was focused on the universe as a whole. {BREAK
0.2} He studied our galaxy, {BREAK 0.2} the Milky Way, {BREAK 0.2}
which consists of ten billion stars, space dust, and gas. {BREAK
0.2} In the 1920s, he proved that there are millions of other
galaxies, and that the distance between them changes according to
certain rules. {BREAK 0.2}

      This break-through, combined with his further discoveries,
{BREAK 0.1} overthrew the previous theory of a static, or
unchanging, universe, {BREAK 0.2} and made Edwin Hubble the founder
of modern cosmology. {BREAK 0.2} His investigations and publications
brought him immense recognition, even among ordinary people. {BREAK
0.2} Despite this recognition, the Nobel Prize was still only a
dream for Edwin Hubble at the time of his death in 1953. {BREAK 0.2}

      Besides our galaxy with its stars and planets, {BREAK 0.2}
only three other galaxies can be seen without the use of a
telescope. {BREAK 0.2} More can be seen from observatories, {BREAK
0.2} but it is still not enough for many researchers. {BREAK 0.2}
For this reason, {BREAK 0.2} on April 24, 1990, {BREAK 0.2} almost a
hundred years after Hubble had been born, {BREAK 0.2} the National
Aeronautics and Space Administration (NASA) launched a huge
telescope into space, which they named after him. {BREAK 0.2}

      The Hubble Space Telescope is constructed from large mirrors
and lenses that must be perfectly set, {BREAK 0.1} a camera, {BREAK
0.1} and several satellites transmitting images of distant objects.
{BREAK 0.2} It helps scientists to study these objects and to
understand processes in the universe.
    </template>
  </category>

  <category>
```

```
    <pattern>STXPG ONE</pattern>
    <template>
      {EMOTE blink 0.3 neutral 1 brows smallhappy neutral 1 leftsg
0.7} American astronomer Edwin Hubble was born in
      November 1889 in Montana. {BREAK} {EMOTE downeyes 0.8 neutral
1.2 leftbrow neutral 1.8 wonder neutral 2 smallhappy 2 | neutral 7
upqg 1}
      After a few years' study at the University of Chicago, he was
awarded a scholarship to Oxford University in Great Britain
      for his excellent athletic and academics skills. {BREAK}
    </template>
  </category>

  <category>
    <pattern>STXPG TWO</pattern>
    <template>
      {EMOTE absent neutral 1.5 upsg 0.4 | neutral 0.5 frust} As he
had promised his father, Edwin concentrated on studying law and
foreign languages rather
      than science. {BREAK 0.2} {EMOTE neutal 0.3 rightglance 0.5 |
neutral 0.8 leftqg 0.2 neutral 0.8 brows} When he returned to the
United States, he became a teacher
      of Spanish. {BREAK 0.2} {EMOTE bliss neutral 0.2 grin}
However, his love was astronomy. {BREAK 0.2}
    </template>
  </category>

  <category>
    <pattern>STXPG THREE</pattern>
    <template>
      {EMOTE downeyes 0.5 | sad 2 brows neutral 1 wonder} After
World War one, he accepted an offer to work in the
      prestigious Mount Wilson Observatory. {BREAK 0.2} {EMOTE
leftbrow 0.5 | smallhappy 0.3 neutral 1 rightqg 1 neutral 1 bigeyes
0.3}
      Ambitious and energetic, he pursued his career and became the
most significant astronomer in the history of cosmology.
      {BREAK} {EMOTE happy} {BREAK}
    </template>
  </category>

  <category>
    <pattern>STXPG FOUR</pattern>
    <template>
      {EMOTE neutral 3 surprise} His research was focused on the
universe as a whole. {BREAK 0.2} {EMOTE upeyes}
      He studied our galaxy, {BREAK 0.2} {EMOTE smallhappy} the
Milky Way, {BREAK 0.2} {EMOTE neutral 0.5 wonder 0.5 neutral 0.8
blink 0.8}
      which consists of ten billion stars, space dust, and gas.
{BREAK 0.2}
      {EMOTE neutral 4 leftglance 0.1 rightglance 0.1 | neutral 4
upeyes neutral 1 rightbrow 1 neutral 1 leftqg 1} In the 1920s, he
proved that there are millions
```

```
      of other galaxies, and that the distance between them changes
according to certain rules. {BREAK 0.2}
    </template>
  </category>

  <category>
    <pattern>STXPG FIVE</pattern>
    <template>
      {EMOTE wonder downqg} This break-through, combined with his
further discoveries, {BREAK 0.1}
      {EMOTE xyeahright | neutral 0.8 blink 0.2} overthrew the
previous theory of a static, or unchanging, universe, {BREAK 0.2}
      {EMOTE leftqg 1 | neutral 2.5 grin} and made Edwin Hubble the
founder of modern cosmology. {BREAK 0.2}
      {EMOTE neutral 0.5 rightqg 0.7 neutral 0.5 blink 1 | brows
neutral 2.2 surprise} His investigations and publications brought
him immense
      recognition, even among ordinary people. {BREAK 0.2} {EMOTE
leftbrow 1 neutral 0.5 absent 3 | neutral 1 bigsad 2 neutral 3
insulted 1}
      Despite this recognition, the Nobel Prize was still only a
dream for Edwin Hubble at the time of his death in 1953. {BREAK 0.2}
    </template>
  </category>

  <category>
    <pattern>STXPG SIX</pattern>
    <template>
      {EMOTE neutral 0.3 upnod 1 | neutral 0.2 brows 0.5} Besides
our galaxy with its stars and planets, {BREAK 0.2}
      {EMOTE rightbrow neutral 1 leftsg 0.4} only three other
galaxies can be seen without the use of a telescope.
      {BREAK 0.2} {EMOTE upeyes 0.3 | brows} More can be seen from
observatories, {BREAK 0.2} {EMOTE neutral 0.5 downqg 1}
      but it is still not enough for many researchers. {BREAK 0.2}
For this reason, {BREAK 0.2} {EMOTE brows} on April 24, 1990,
      {BREAK 0.2} {EMOTE neutral 2 leftbrow | neutral 1.5 rightsg
0.2} almost a hundred years after Hubble had been born,
      {BREAK 0.2} {EMOTE neutral 5.4 surprise} the National
Aeronautics and Space Administration (NASA) launched a huge
      telescope into space, which they named after him. {BREAK}
{EMOTE grin} {BREAK}
    </template>
  </category>

  <category>
    <pattern>STXPG SEVEN</pattern>
    <template>
      {EMOTE brows neutral 1 rightglance 0.3 neutral 4 bigeyes 0.2}
The Hubble Space Telescope is constructed
      from large mirrors and lenses that must be perfectly set,
{BREAK 0.1} a camera, {BREAK 0.1}
      {EMOTE neutral 0.5 wonder | neutral 0.2 upsg 0.3} and several
satellites transmitting images of distant
```

```
      objects. {BREAK 0.2} {EMOTE happy neutral 0.5 brows} It helps
scientists to study these objects and
      to understand processes in the universe. {BREAK 0.2} {EMOTE
happy}
    </template>
  </category>

  <category>
    <pattern>STXPGH</pattern>
    <template>
      <srai>STXPG ONE</srai>
      <srai>STXPG TWO</srai>
      <srai>STXPG THREE</srai>
      <srai>STXPG FOUR</srai>
      <srai>STXPG FIVE</srai>
      <srai>STXPG SIX</srai>
      <srai>STXPG SEVEN</srai>
    </template>
  </category>

  <category>
    <pattern>STXPNM</pattern>
    <template>
      Machu Picchu {BREAK 0.1} which means manly peak {BREAK 0.1}
      is an ancient Incan city located high in the Andes mountains
of Peru.
      {BREAK 0.2} It is on top of a ridge between mountains, {BREAK
0.2}
      and is hidden from the Urabamba gorge below. {BREAK 0.2}
      The mighty mountain Huaynac Picchu towers above, {BREAK 0.2}
      and green jungle surrounds the ancient city. {BREAK 0.2}
      It was built between 1460 and 1470 AD by the Incan ruler
Pachacuti Inca Yupanqui, {BREAK 0.2}
      and was probably a royal estate and religious retreat. {BREAK
0.2}

      There are about two hundred buildings in Machu Picchu. {BREAK
0.2}
      Many of the buildings are homes, {BREAK 0.2} but there are
also many temples
      and other buildings, such as storehouses. {BREAK 0.2} The
buildings are all made of
      granite  blocks that were cut with bronze or stone tools, and
then smoothed with sand. {BREAK 0.2}
      Each block fits perfectly against the next block, {BREAK 0.2}
{EMOTE grin 1} and no mortar
      was used! {BREAK 0.2}

      Several hundred years ago, Machu Picchu was abandoned for an
unknown reason. {BREAK 0.2}
      With civil war within the Incan Empire, {BREAK 0.2} massive
deaths
      because of European diseases, {BREAK 0.2}  and the Spanish
invasion, {BREAK 0.2}
```

```
      Machu Picchu was soon forgotten, {BREAK 0.2} and even Pizarro,
{BREAK 0.2}
      the man who finally conquered the Incas, {BREAK 0.2}
      probably never knew about Machu Picchu.  {BREAK 0.2}

      Machu Picchu remained untouched for about four hundred years,
      {BREAK 0.2}  before it was rediscovered by Hiram Bingham in
1911.
      {BREAK 0.2} Mr. Bingham was originally looking for Vilcabamba,
{BREAK 0.2}
      the last undiscovered stronghold of the Incan Empire. {BREAK
0.2}
      When he found Machu Picchu, {BREAK 0.2}  he believed it
      was Vilcabamba. {BREAK 0.2}  For several years, Machu Picchu
was thought to be Vilcabamba,
      {BREAK 0.2} but most people now believe Machu Picchu was an
entirely different city.
    </template>
  </category>

  <category>
    <pattern>STXPGM ONE</pattern>
    <template>
      {EMOTE blink 0.3} Machu Picchu {BREAK 0.1} {EMOTE neutral 0.2
downeyes 0.7} which means manly peak {BREAK 0.1}
      {EMOTE wonder neutral 0.5 brows 0.5 | neutral 3 upqg 1} is an
ancient Incan city located high in the Andes mountains of Peru.
      {BREAK 0.2} {EMOTE neutral 0.8 upnod 0.5} It is on top of a
ridge between mountains, {BREAK 0.2}
      {EMOTE neutral 0.2 blink 1.5} and is hidden from the Urabamba
gorge below. {BREAK 0.2} {EMOTE neutral 0.5 wonder 1 | neutral 0.7
downqg 1.5}
      The mighty mountain Huaynac Picchu towers above, {BREAK 0.2}
{EMOTE neutral 0.2 halfblink 1.5 smallhappy | neutral 2 leftqg 1.2}
      and green jungle surrounds the ancient city. {BREAK 0.2}
{EMOTE neutral 1 brows 1 neutral 1 downeyes 2 | neutral 5.5 bigeyes}
      It was built between 1460 and 1470 AD by the Incan ruler
Pachacuti Inca Yupanqui, {BREAK 0.2} {EMOTE neutral 0.8 wonder 1.1
happy 0.3}
      and was probably a royal estate and religious retreat. {BREAK
0.2}
    </template>
  </category>

  <category>
    <pattern>STXPGM TWO</pattern>
    <template>
      {EMOTE neutral 1 rightbrow 0.5} There are about two hundred
buildings in Machu Picchu. {BREAK 0.2} {EMOTE wonder 1}
      Many of the buildings are homes, {BREAK 0.2} {EMOTE neutral 1
upsg 0.5 | neutral 2.5 swonder downqg 1} but there are also many
temples
      and other buildings, such as storehouses. {BREAK 0.2} {EMOTE
neutral 2 brows 0.5 neutral 1 downqg 1 | leftbrow 0.5 neutral 1.5
```

```
frust leftqg 0.5 rightqg 0.5}
      The buildings are all made of granite  blocks that were cut
with bronze or stone tools, and then smoothed with sand. {BREAK 0.2}
      {EMOTE neutral 0.5 wonder 2} Each block fits perfectly against
the next block, {BREAK 0.2} {EMOTE grin 1} and no mortar
      was used! {BREAK 0.2}
    </template>
  </category>

  <category>
    <pattern>STXPGM THREE</pattern>
    <template>
      {EMOTE blink 0.6 neutral 1 brows 1 leftsg 0.3} Several hundred
years ago, Machu Picchu was abandoned for an unknown reason. {BREAK
0.2}
      {EMOTE sad} With civil war within the Incan Empire, {BREAK
0.2} {EMOTE bigeyes | frust} massive deaths
      because of European diseases, {BREAK 0.2} {EMOTE neutral 0.1
anger} and the Spanish invasion, {BREAK 0.2} {EMOTE downeyes 1.6}
      Machu Picchu was soon forgotten, {BREAK 0.2} {EMOTE brows} and
even Pizarro, {BREAK 0.2}
      {EMOTE insulted 0.8 | downqg 1} the man who finally conquered
the Incas, {BREAK 0.2} {EMOTE leftqg 2 | neutral 1 downnod 1}
      probably never knew about Machu Picchu.  {BREAK 0.2}
    </template>
  </category>

  <category>
    <pattern>STXPGM FOUR</pattern>
    <template>
      {EMOTE leftbrow 0.8 | neutral 0.4 smallhappy} Machu Picchu
remained untouched for about four hundred years,
      {BREAK 0.2} {EMOTE neutral 2 brows 1 | neutral 1 rightqg 1}
before it was rediscovered by Hiram Bingham in 1911.
      {BREAK} {EMOTE grin} {BREAK}
      {EMOTE neutral 2 leftqg 1 | neutral 2 upqg 1} Mr. Bingham was
originally looking for Vilcabamba, {BREAK 0.2}
      {EMOTE neutral 0.5 wonder 1} the last undiscovered stronghold
of the Incan Empire. {BREAK 0.2}
      {EMOTE rightbrow 0.1} When he found Machu Picchu, {BREAK 0.2}
{EMOTE smile | leftqg 1} he believed it
      was Vilcabamba. {BREAK 0.2} {EMOTE wonder | neutral 1 upsg
0.5} For several years, Machu Picchu was thought to be Vilcabamba,
      {BREAK 0.2} {EMOTE neutral 1 downsg 0.5 | neutral 3 brows} but
most people now believe Machu Picchu was an entirely different city.
      {BREAK} {EMOTE smallhappy}
    </template>
  </category>

  <category>
    <pattern>STXPGM</pattern>
    <template>
      <srai>STXPGM ONE</srai>
      <srai>STXPGM TWO</srai>
```

```
      <srai>STXPGM THREE</srai>
      <srai>STXPGM FOUR</srai>
    </template>
  </category>

  <category>
    <pattern>STXPRH</pattern>
    <template>
      {EMOTE random 0.3 neutral 1 random random neutral 1.8 random
0.25} American astronomer Edwin Hubble was born in
      November 1889 in Montana. {BREAK} {EMOTE random 0.8 neutral
1.2 random neutral 1.8 random neutral 2 random 2 | neutral 7 random
1}
      After a few years' study at the University of Chicago, he was
awarded a scholarship to Oxford University in Great Britain
      for his excellent athletic and academics skills. {BREAK}

      {EMOTE random neutral 1.5 random 0.4 | neutral 0.5 random} As
he had promised his father, Edwin concentrated on studying law and
foreign languages rather
      than science. {BREAK 0.2} {EMOTE neutal 0.3 random 0.5 |
neutral 0.8 random 0.2 neutral 0.8 random} When he returned to the
United States, he became a teacher
      of Spanish. {BREAK 0.2} {EMOTE random neutral 0.2 random}
However, his love was astronomy. {BREAK 0.2}

      {EMOTE random 0.5 | neutral 2 random neutral 1 random} After
World War one, he accepted an offer to work in the
      prestigious Mount Wilson Observatory. {BREAK 0.2} {EMOTE
random 0.5 | random 0.3 neutral 1 random 1 neutral 1 random 0.3}
      Ambitious and energetic, he pursued his career and became the
most significant astronomer in the history of cosmology.
      {BREAK} {EMOTE random} {BREAK}

      {EMOTE neutral 3 random} His research was focused on the
universe as a whole. {BREAK 0.2} {EMOTE random}
      He studied our galaxy, {BREAK 0.2} {EMOTE random} the Milky
Way, {BREAK 0.2} {EMOTE neutral 0.5 random 0.5 neutral 0.8 random
0.8}
      which consists of ten billion stars, space dust, and gas.
{BREAK 0.2}
      {EMOTE neutral 4 random 0.1 random 0.1 | neutral 4 random
neutral 1 random 1 neutral 1 random 1} In the 1920s, he proved that
there are millions
      of other galaxies, and that the distance between them changes
according to certain rules. {BREAK 0.2}

      {EMOTE random random} This break-through, combined with his
further discoveries, {BREAK 0.1}
      {EMOTE random | neutral 0.8 random 0.2} overthrew the previous
theory of a static, or unchanging, universe, {BREAK 0.2}
      {EMOTE random 1 | neutral 2.5 random} and made Edwin Hubble
the founder of modern cosmology. {BREAK 0.2}
      {EMOTE neutral 0.5 random 0.7 neutral 0.5 random 1 | random
```

```
neutral 2.2 random} His investigations and publications brought him
immense
      recognition, even among ordinary people. {BREAK 0.2} {EMOTE
random 1 neutral 0.5 random 3 | neutral 1 random 2 neutral 3 random
1}
      Despite this recognition, the Nobel Prize was still only a
dream for Edwin Hubble at the time of his death in 1953. {BREAK 0.2}

      {EMOTE neutral 0.3 random 1 | neutral 0.2 random 0.5} Besides
our galaxy with its stars and planets, {BREAK 0.2}
      {EMOTE random neutral 1 random 0.2} only three other galaxies
can be seen without the use of a telescope.
      {BREAK 0.2} {EMOTE random 0.3 | random} More can be seen from
observatories, {BREAK 0.2} {EMOTE neutral 0.5 random 1}
      but it is still not enough for many researchers. {BREAK 0.2}
For this reason, {BREAK 0.2} {EMOTE random} on April 24, 1990,
      {BREAK 0.2} {EMOTE neutral 2 random | neutral 1.5 random 0.2}
almost a hundred years after Hubble had been born,
      {BREAK 0.2} {EMOTE neutral 5.4 random} the National
Aeronautics and Space Administration (NASA) launched a huge
      telescope into space, which they named after him. {BREAK}
{EMOTE random} {BREAK}

      {EMOTE random neutral 1 random 0.3 neutral 4 random 0.2} The
Hubble Space Telescope is constructed
      from large mirrors and lenses that must be perfectly set,
{BREAK 0.1} a camera, {BREAK 0.1}
      {EMOTE neutral 0.5 random | neutral 0.2 random 0.3} and
several satellites transmitting images of distant
      objects. {BREAK 0.2} {EMOTE random neutral 0.5 random} It
helps scientists to study these objects and
      to understand processes in the universe. {BREAK 0.2} {EMOTE
random}

    </template>
  </category>

  <category>
    <pattern>STXPT</pattern>
    <template>
      {EMOTE wonder} Thank you for your participation in this
experiment. {BREAK} {EMOTE wink}
    </template>
  </category>
```
